# ANGPTL3 negatively regulates IL-1**β**-induced NF-**κ**B activation by inhibiting the IL1R1-associated signaling complex assembly

**DOI:** 10.1093/jmcb/mjad053

**Published:** 2023-08-26

**Authors:** Yu Zhang, Zi-tong Zhang, Shi-yuan Wan, Jing Yang, Yu-juan Wei, Hui-jing Chen, Wan-zhu Zhou, Qiu-yi Song, Shu-xuan Niu, Ling Zheng, Kun Huang

**Affiliations:** Tongji School of Pharmacy, Tongji Medical College and State Key Laboratory for Diagnosis and Treatment of Severe Zoonotic Infectious Diseases, Huazhong University of Science and Technology, Wuhan 430030, China; Tongji School of Pharmacy, Tongji Medical College and State Key Laboratory for Diagnosis and Treatment of Severe Zoonotic Infectious Diseases, Huazhong University of Science and Technology, Wuhan 430030, China; Tongji School of Pharmacy, Tongji Medical College and State Key Laboratory for Diagnosis and Treatment of Severe Zoonotic Infectious Diseases, Huazhong University of Science and Technology, Wuhan 430030, China; Tongji School of Pharmacy, Tongji Medical College and State Key Laboratory for Diagnosis and Treatment of Severe Zoonotic Infectious Diseases, Huazhong University of Science and Technology, Wuhan 430030, China; Tongji School of Pharmacy, Tongji Medical College and State Key Laboratory for Diagnosis and Treatment of Severe Zoonotic Infectious Diseases, Huazhong University of Science and Technology, Wuhan 430030, China; Tongji School of Pharmacy, Tongji Medical College and State Key Laboratory for Diagnosis and Treatment of Severe Zoonotic Infectious Diseases, Huazhong University of Science and Technology, Wuhan 430030, China; Tongji School of Pharmacy, Tongji Medical College and State Key Laboratory for Diagnosis and Treatment of Severe Zoonotic Infectious Diseases, Huazhong University of Science and Technology, Wuhan 430030, China; Tongji School of Pharmacy, Tongji Medical College and State Key Laboratory for Diagnosis and Treatment of Severe Zoonotic Infectious Diseases, Huazhong University of Science and Technology, Wuhan 430030, China; Tongji School of Pharmacy, Tongji Medical College and State Key Laboratory for Diagnosis and Treatment of Severe Zoonotic Infectious Diseases, Huazhong University of Science and Technology, Wuhan 430030, China; Hubei Key Laboratory of Cell Homeostasis, College of Life Sciences, Wuhan University, Wuhan 430072, China; Tongji School of Pharmacy, Tongji Medical College and State Key Laboratory for Diagnosis and Treatment of Severe Zoonotic Infectious Diseases, Huazhong University of Science and Technology, Wuhan 430030, China

**Keywords:** inflammation, NF-κB, IL-1β, IL1R1, intracellular ANGPTL3

## Abstract

Interleukin-1β (IL-1β)-induced signaling is one of the most important pathways in regulating inflammation and immunity. The assembly of the receptor complex, consisting of the ligand IL-1β, the IL-1 receptor (IL-1R) type 1 (IL1R1), and the IL-1R accessory protein (IL1RAP), initiates this signaling. However, how the IL1R1-associated complex is regulated remains elusive. Angiopoietin like 3 (ANGPTL3), a key inhibitor of plasma triglyceride clearance, is mainly expressed in the liver and exists in both intracellular and extracellular secreted forms. Currently, ANGPTL3 has emerged as a highly promising drug target for hypertriglyceridemia and associated cardiovascular diseases. However, most studies have focused on the secreted form of ANGPTL3, while its intracellular role is still largely unknown. Here, we report that intracellular ANGPTL3 acts as a negative regulator of IL-1β-triggered signaling. Overexpression of ANGPTL3 inhibited IL-1β-induced NF-κB activation and the transcription of inflammatory genes in HepG2, THP1, and HEK293T cells, while knockdown or knockout of ANGPTL3 resulted in opposite effects. Mechanistically, ANGPTL3 interacted with IL1R1 and IL1RAP through its intracellular C-terminal fibrinogen-like domain and disrupted the assembly of the IL1R1-associated complex. Taken together, our study reveals a novel role for ANGPTL3 in inflammation, whereby it inhibits the physiological interaction between IL1R1 and IL1RAP to maintain immune tolerance and homeostasis in the liver.

## Introduction

Interleukin-1 (IL-1), including IL-1α and IL-1β, are master inflammatory cytokines that play an important role in inflammatory responses and autoimmune diseases (Dinarello, 2009). The IL-1 receptor (IL-1R) type 1 (IL1R1), a member of the Toll/IL-1R (TIR) homology domain-containing receptor family, is expressed in almost all types of cells. Upon the detection of infectious stimuli or endogenous sterile danger signals (e.g. high concentrations of extracellular ATP, crystals of cholesterol, etc.), inflammasome-cleaved IL-1 binds to IL1R1, thus modulating the immunological microenvironment and restoring homeostasis (Boraschi et al., 2018).

When stimulated by IL-1α and IL-1β, the cell membrane IL1R1 associates with the IL-1R accessory protein (IL1RAP) to form a trimeric IL-1R complex. This IL-1R complex recruits myeloid differentiation primary response protein 88 (MyD88), which subsequently interacts with several IL-1R-associated kinases (IRAKs) as well as the E3 ubiquitin ligase TRAF6, forming a signaling scaffold known as the myddosome (Greenfeder et al., 1995; Lin et al., 2010; Luchini et al., 2014). The myddosome activates the downstream kinase TAK1, which in turn activates IKKs, resulting in the activation of nuclear factor-κB (NF-κB) and the initiation of inflammatory responses (Dinarello, 2009; Boraschi et al., 2018). Due to the critical roles of IL-1β-induced signaling in regulating inflammation and immunity, the IL-1β-triggered pathway is tightly controlled through various mechanisms. For example, the IL-1R antagonist (IL1Ra), a molecule structurally similar to IL-1, has a high affinity for IL1R1 (Schreuder et al., 1997). Furthermore, certain receptors, such as IL1R2, IL1R8, and SIGIRR, act as decoy receptors, capturing and preventing IL-1 binding to IL1R1 (Boraschi et al., 2018). In addition, MARCH3 and MARCH8 are responsible for the degradation of IL1R1 and IL1RAP, respectively (Chen et al., 2012; Lin et al., 2018).

The liver is constantly exposed to circulating antigens and endotoxins derived from the gut microbiota, making tightly controlled immune responses and inflammation crucial for its homeostasis maintenance (Heymann and Tacke, 2016). Accumulating evidence suggests the importance of IL-1β–IL1R1 signaling in liver homeostasis. For example, hepatocyte-specific deletion of IL1R1 attenuates liver injury (Gehrke et al., 2018), while IL1Ra ameliorates inflammasome-dependent alcoholic steatohepatitis, decreases liver fibrosis, and improves liver regeneration in mouse models (Petrasek et al., 2012; Zhou et al., 2020b). Clinical trials using recombinant IL1Ra (anakinra) have shown benefits in treating severe alcoholic steatohepatitis (Szabo et al., 2022). Despite these findings, the precise regulatory mechanisms of this signaling pathway in the liver remain elusive.

Angiopoietin like 3 (ANGPTL3) is a member of the ANGPTL family, which consists of eight secreted proteins that are homologous to angiopoietins. The ANGPTL family proteins are involved in the regulation of angiogenesis, lipid metabolism, inflammation, and cancer progression (Carbone et al., 2018; Yang et al., 2022). Among these ANGPTLs, ANGPTL3 is mainly expressed in the liver and serves as a key regulator of plasma lipoproteins. Its circulating form inhibits the activity of lipoprotein lipase (LPL), leading to increased serum triglycerides (TG) (Kersten, 2017). Structurally, ANGPTL3 consists of an N-terminal signal peptide, two coiled-coil domains (CCDs), and a C-terminal fibrinogen-like domain (FLD). The FLD, which is highly conserved within the ANGPTL family, is associated with tumor pathogenesis (Wang et al., 2018; Yang et al., 2022). Physiologically, glycosylated ANGPTL3 undergoes cleavage, generating intracellular and extracellular N-terminal and C-terminal fragments separated by ^221^RAPR^224^ (Ono et al., 2003; Essalmani et al., 2013). The N-terminus of ANGPTL3 is important for lipid metabolism, while the C-terminal FLD domain is involved in angiogenesis (Camenisch et al., 2002).

The significance of ANGPTL3 has been supported by multiple *in vitro* and *in vivo* studies, and clinical findings further reinforced its importance. Compound heterozygous loss-of-function mutations in ANGPTL3 cause familial combined hypolipidemia, a genetic disease characterized by low plasma levels of low-density lipoproteins, high-density lipoproteins, and TG (Musunuru et al., 2010; Minicocci et al., 2012; Pisciotta et al., 2012). Given its role in dyslipidemia, ANGPTL3 has become one of the most promising drug targets, especially for patients who do not benefit from statins (Kersten, 2017). An ANGPTL3 monoclonal antibody (mAb, evinacumab) was approved by the FDA in 2021 for the treatment of homozygous familial hypercholesterolemia (Ruscica et al., 2020; Mullard, 2021). Additionally, more ANGPTL3-targeted therapies, including antisense oligonucleotides, RNAi, small molecular lead compounds, and gene editing, are under development (Yang et al., 2022). Although there are mAbs specifically targeting circulating ANGPTL3 and other antagonists targeting both intracellular and extracellular ANGPTL3, the functions of intracellular ANGPTL3 remain poorly understood, which limits the development of ANGPTL3-based therapies. Previously, we identified ANGPTL8, another member of the ANGPTL family, as a negative intracellular regulator of NF-κB activation and inflammation (Zhang et al., 2017). In this study, we provide evidence that intracellular ANGPTL3 acts as a negative regulator of IL-1β- but not lipopolysaccharide (LPS)-induced inflammatory response via disassembling the IL1R1-associated complex. Our findings uncover a new mechanism for NF-κB regulation and expand our understanding of the roles of intracellular ANGPTL3 in the liver.

## Results

### Overexpression of ANGPTL3 inhibits IL-1β-induced NF-κB activation

**Figure 1:**
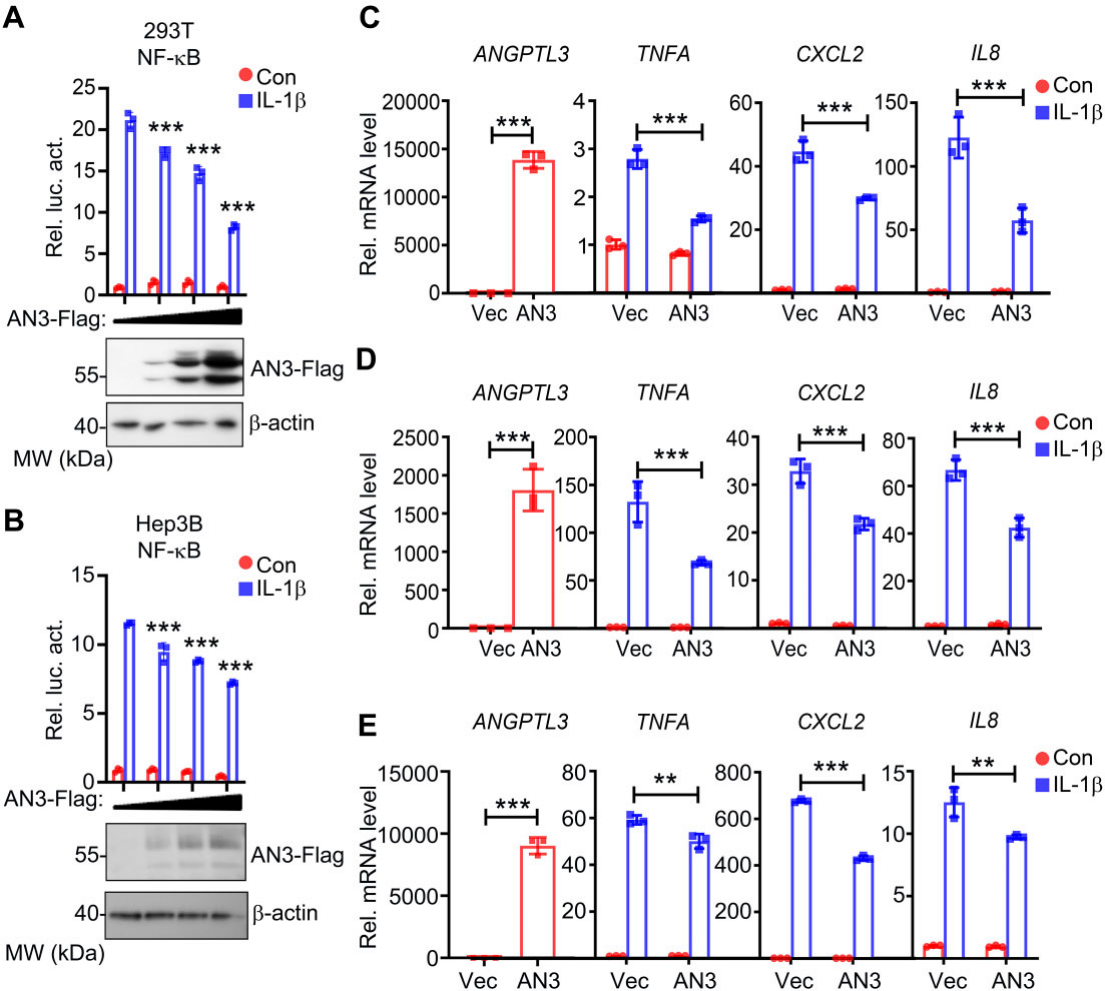
Overexpression of ANGPTL3 inhibits IL-1β-induced NF-κB activation. (**A** and **B**) Overexpression of ANGPTL3 inhibits IL-1β-induced NF-κB activation in a dose-dependent manner as determined by the reporter assay in HEK293T (upper panel in **A**) and Hep3B (upper panel in **B**) cells. The expression levels of ANGPTL3-Flag in the indicated cell lysate were detected by western blotting (lower panels). (**C**–**E**) The effects of overexpressed ANGPTL3 on IL-1β-induced transcription of *TNFA, CXCL2*, and *IL8* were determined by qPCR in HEK293T (**C**), Hep3B (**D**), and primary hepatocytes (**E**). AN3, ANGPTL3. **P < 0.01; ***P < 0.001.

To explore the role of ANGPTL3 in IL-1β-induced NF-κB activation, we overexpressed ANGPTL3-Flag plasmids in the human embryonic kidney cell line HEK293T and the human hepatocellular carcinoma cell line Hep3B. Reporter assay results suggested that overexpression of ANGPTL3 inhibited IL-1β-induced NF-κB activation in a dose-dependent manner (Figure 1A and B). Similarly, the overexpression of ANGPTL3 attenuated IL-1β-induced transcription of *TNFA, CXCL2*, and *IL8*, three downstream effector genes of NF-κB, in both HEK293T and Hep3B cells (Figure 1C and D). Moreover, ANGPTL3 overexpression inhibited IL-1β-induced upregulation of NF-κB downstream genes in primary mouse hepatocytes (Figure 1E). These results collectively indicate that ANGPTL3 plays an inhibitory role in IL-1β-induced signaling pathway.

### Knockdown or knockout of ANGPTL3 potentiates IL-1β-induced NF-κB activation

**Figure 2:**
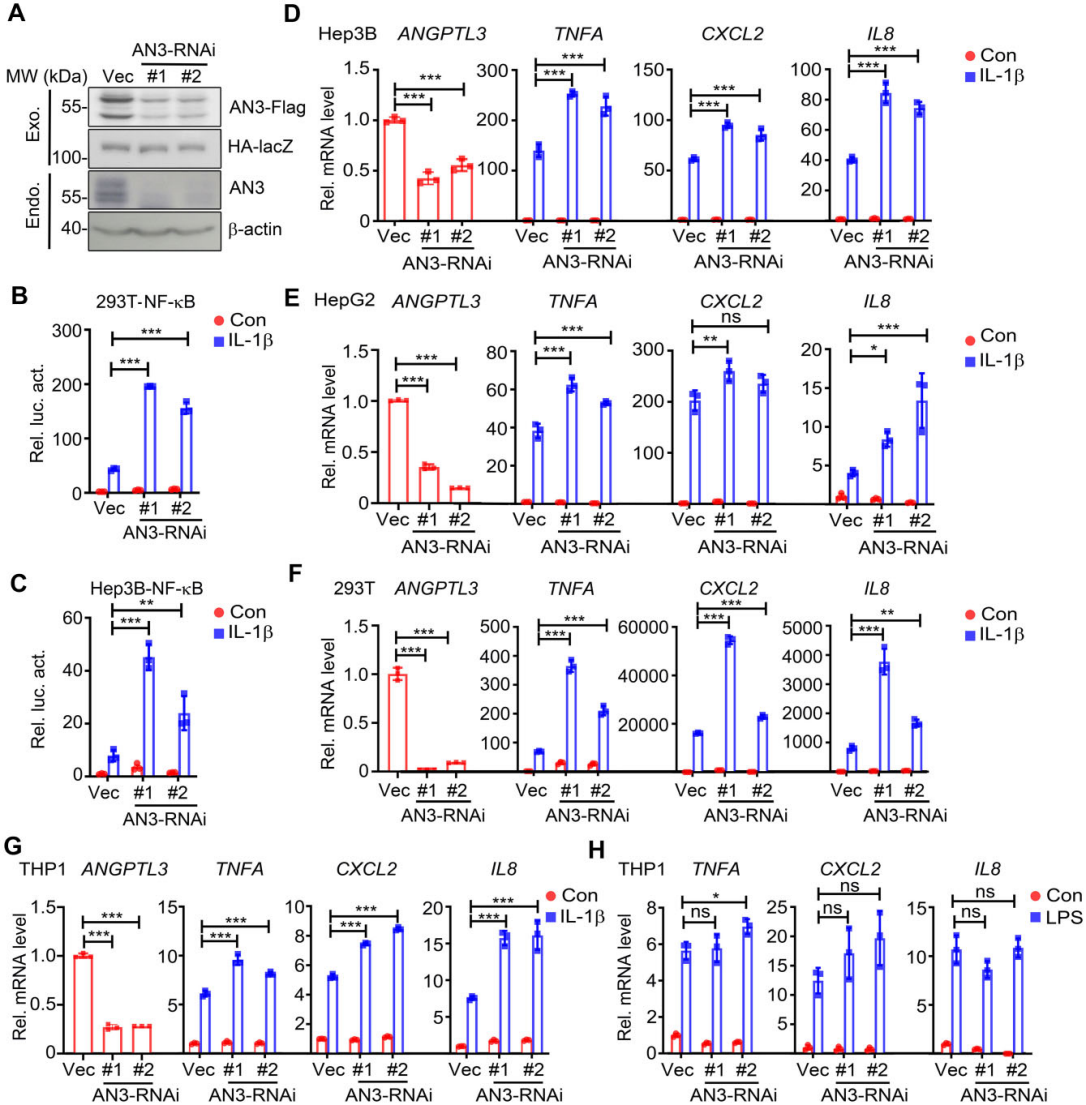
Knockdown of ANGPTL3 potentiates IL-1β-induced NF-κB activation. (**A**) ANGPTL3 shRNA plasmids significantly reduced the protein levels of both transfected Flag-tagged ANGPTL3 (upper panel, in HEK293T cells) and endogenous ANGPTL3 (lower panel, in Hep3B cells). (**B** and **C**) The effects of ANGPTL3 knockdown on IL-1β-induced NF-κB activation were determined by the reporter assay in HEK293T (**B**) and Hep3B (**C**) cells. (**D**–**F**) The effects of ANGPTL3 knockdown on IL-1β-induced transcription of *TNFA, CXCL2*, and *IL8* were determined by qPCR in Hep3B (**D**), HepG2 (**E**), and HEK293T (**F**) cells. (**G** and **H**) The effects of ANGPTL3 knockdown on IL-1β- (**G**) or LPS-induced (**H**) transcription of *TNFA, CXCL2*, and *IL8* were determined by qPCR in THP1 cells. AN3, ANGPTL3; Exo., exogenous; Endo., endogenous. *P < 0.05; **P < 0.01; ***P < 0.001; ns, not significant.

To further investigate the functions of ANGPTL3 in IL-1β-induced signaling, we constructed two shRNA plasmids for ANGPTL3, which target the coding sequence of the ANGPTL3 gene. Both shRNAs significantly reduced the protein levels of transfected Flag-tagged ANGPTL3 and endogenous ANGPTL3 (Figure 2A). The reporter assay results indicated markedly enhanced IL-1β-induced NF-κB activation after ANGPTL3 knockdown in both HEK293T and Hep3B cells (Figure 2B and C). Consistently, knockdown of ANGPTL3 potentiated the transcription of NF-κB downstream effector genes in Hep3B, HepG2, and HEK293T cells (Figure 2D–F). We next determined the effects of ANGPTL3 on NF-κB activation in immune cells by knocking down ANGPTL3 in THP1 cells, a human monocyte cell line from acute monocytic leukemia, and confirmed that the knockdown of ANGPTL3 in THP1 cells also potentiated IL-1β-induced transcription of downstream genes (Figure 2G).

Toll like receptor 4 (TLR4) shares a similar TIR intracellular domain with IL1R1 and is able to recognize microbial patterns (e.g. LPS) to mediate local or systemic antimicrobial host defense (Fitzgerald and Kagan, 2020). Although having different ligands and receptors/co-receptors, the IL-1–IL1R1- and LPS–TLR4-mediated signaling share common downstream pathways. Notably, knockdown of ANGPTL3 did not affect LPS–TLR4-induced transcription of NF-κB downstream genes (Figure 2H), suggesting that ANGPTL3 targets IL-1β- but not LPS-induced NF-κB activation.

**Figure 3:**
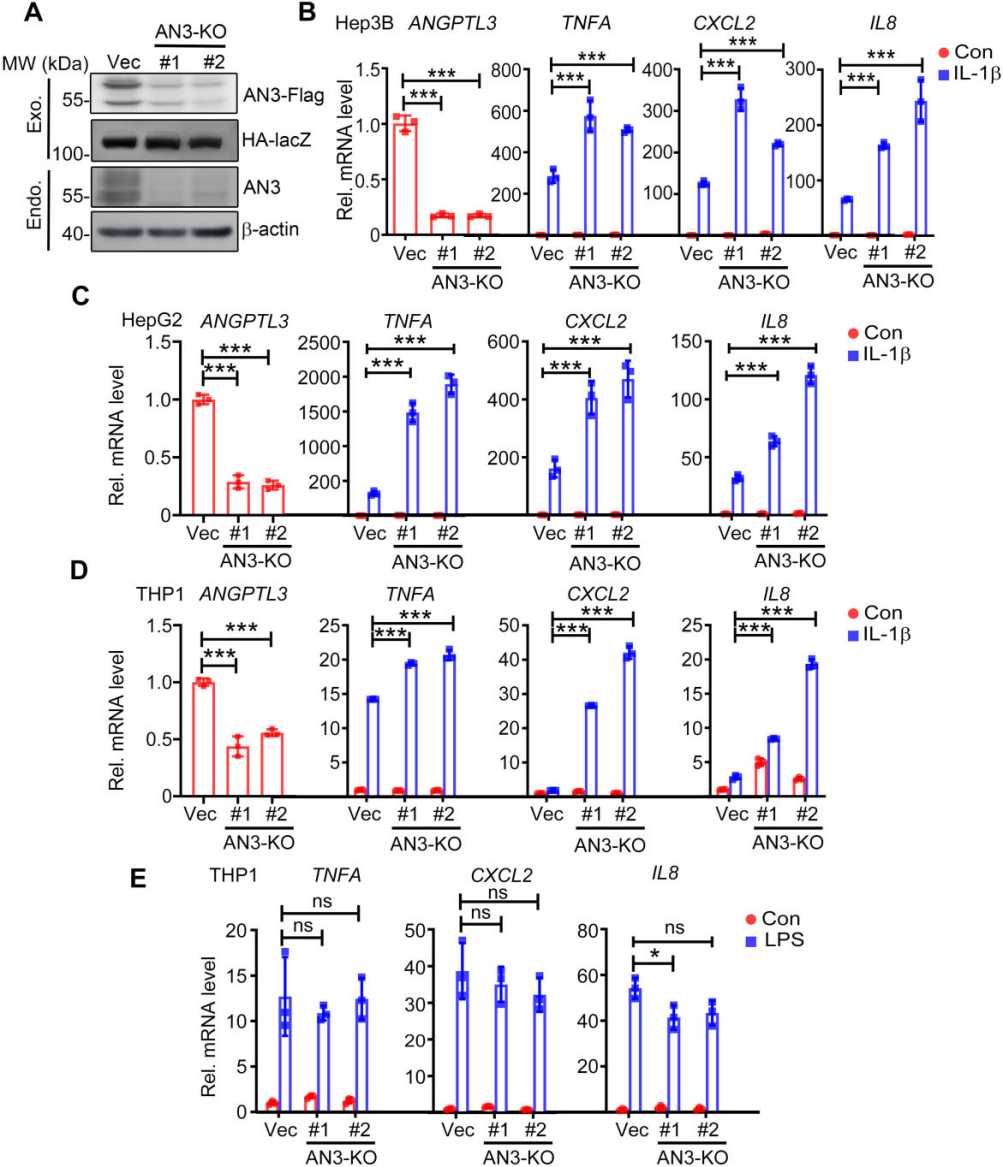
Knockout of ANGPTL3 potentiates IL-1β-induced NF-κB activation. (**A**) ANGPTL3 sgRNA plasmids significantly reduced the protein levels of both transfected Flag-tagged ANGPTL3 (upper panel, in HEK293T cells) and endogenous ANGPTL3 (lower panel, in Hep3B cells). (**B** and **C**) The effects of ANGPTL3 deficiency on IL-1β-induced transcription of *TNFA, CXCL2*, and *IL8* were determined by qPCR in Hep3B (**B**) and HepG2 (**C**) cells. (**D** and **E**) The effects of ANGPTL3 deficiency on IL-1β- (**D**) and LPS-induced (**E**) transcription of *TNFA, CXCL2*, and *IL8* were determined by qPCR in THP1 cells. AN3, ANGPTL3; KO, knockout; Exo., exogenous; Endo., endogenous. *P < 0.05; ***P < 0.001; ns, not significant.

To further examine whether endogenous ANGPTL3 regulates IL-1β-triggered NF-κB activation, we constructed single-guide RNA (sgRNA) plasmids targeting different regions of the coding sequences of the ANGPTL3 gene based on the CRISPR–Cas9 system. The results suggested that these sgRNAs effectively suppressed the expression of transfected Flag-tagged ANGPTL3 in HEK293T cells and decreased endogenous ANGPTL3 expression in Hep3B cells (Figure 3A). Quantitative real-time PCR (qPCR) analysis showed that in Hep3B, HepG2, and THP1 cells, ANGPTL3 deficiency enhanced transcription of NF-κB downstream genes upon IL-1β stimulation (Figure 3B–D); however, LPS-induced transcription of downstream genes was comparable in wild-type and ANGPTL-deficient THP1 cells (Figure 3E). These results further demonstrated that ANGPTL3 plays a negative role in IL-1β- but not LPS-induced NF-κB activation.

### ANGPTL3 targets the IL1R1-associated complex

**Figure 4:**
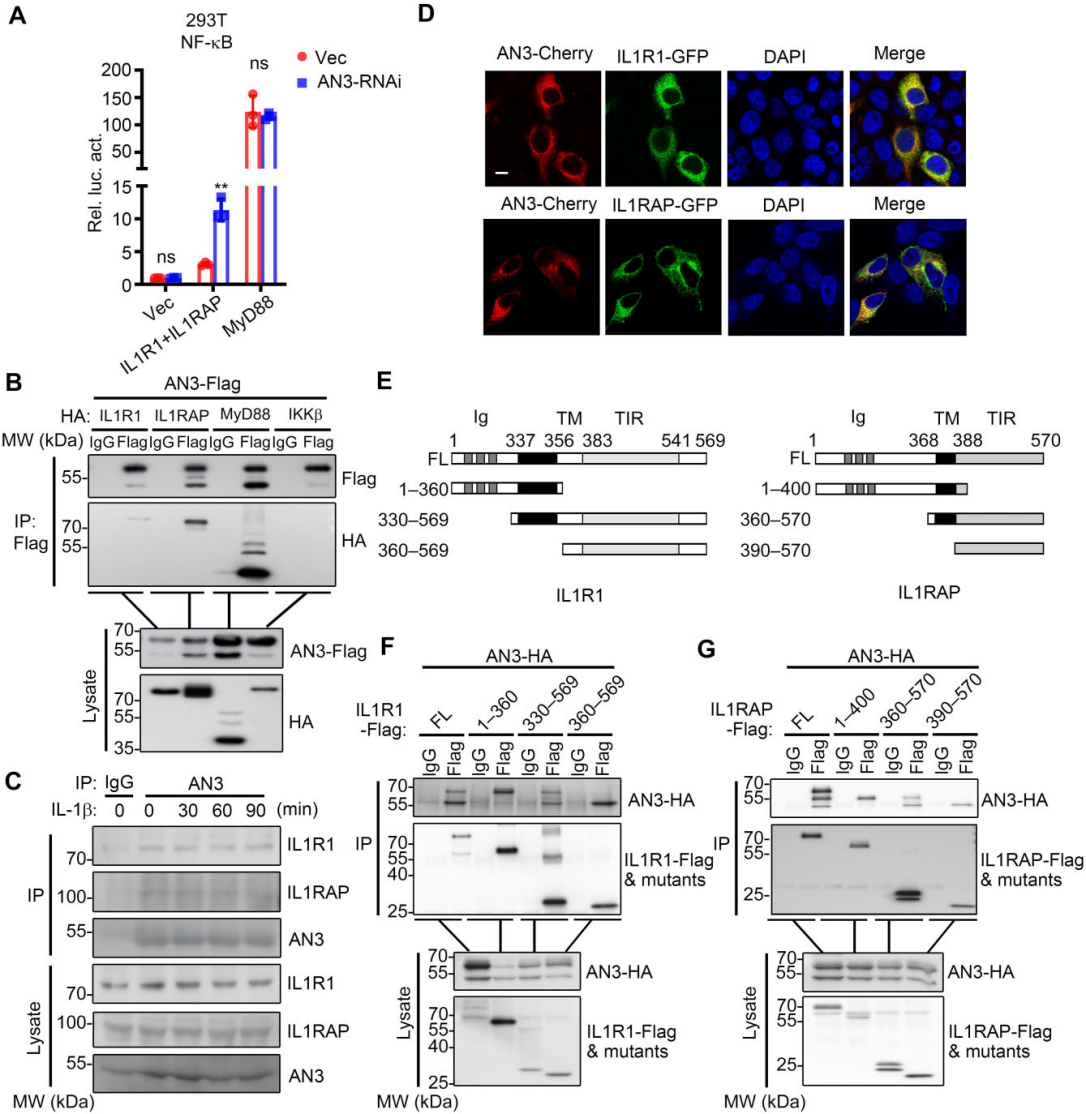
ANGPTL3 targets the IL1R1-associated complex. (**A**) The effects of ANGPTL3 knockdown on IL1R1–IL1RAP- or MyD88-mediated NF-κB activation were determined by the reporter assay in HEK293T cells. **P < 0.01; ns, not significant. (**B**) The interaction of overexpressed ANGPTL3 with IL1R1, IL1RAP, MyD88, and IKKβ was determined by Co-IP in HEK293T cells. (**C**) The interaction of endogenous ANGPTL3 with IL1R1 and IL1RAP was determined by Co-IP in HepG2 cells. (**D**) The co-localization of ANGPTL3-Cherry with IL1R1-GFP and IL1RAP-GFP in HEK293T cells. Scale bar, 10 μm. (**E**) A schematic presentation of human IL1R1, IL1RAP, and their truncation mutants. (**F** and **G**) The interaction of ANGPTL3 with IL1R1, IL1RAP, and their truncation mutants was determined by Co-IP in HEK293T cells. AN3, ANGPTL3; FL, full-length; Ig, immunoglobulin domain; TM, transmembrane domain; TIR: Toll/IL-1R homology domain.

Both IL-1β- and LPS-induced signaling share the adaptor MyD88 and downstream molecules, whereas ANGPTL3 specifically attenuated IL-1β- but not LPS-induced signaling (Figures 2G and H, 3D and E). Therefore, we proposed that ANGPTL3 may target the IL1R1-associated receptor complex. Interestingly, reporter assay results revealed that knockdown of ANGPTL3 potentiated NF-κB activation mediated by IL1R1–IL1RAP but not MyD88, indicating that ANGPTL3 may target the IL1R1–IL1RAP complex (Figure 4A). Co-immunoprecipitation (Co-IP) results suggested that overexpressed ANGPTL3 interacted with upstream receptors and adaptors, including IL1R1, IL1RAP, and MyD88, but not with the downstream IKKβ (Figure 4B). Furthermore, Co-IP results in HepG2 cells indicated that endogenous ANGPTL3 interacted with IL1R1 and IL1RAP both at the resting stage and under IL-1β treatment (Figure 4C). Consistently, co-localization of ANGPTL3 with IL1R1 and IL1RAP was observed in cell membranes and intracellular structures (Figure 4D).

Next, we constructed a set of truncation mutants of IL1R1 and IL1RAP (Figure 4E) to map their interaction regions with ANGPTL3. IL1R1 and IL1RAP all consist of an extracellular N-terminal immunoglobulin (Ig) domain that mediates ligand binding, a transmembrane domain, and an intracellular C-terminal TIR domain that mediates the IL1R1–IL1RAP interaction and downstream signaling. Co-IP results indicated that ANGPTL3 interacted with both N- and C-terminal regions of IL1R1 and IL1RAP (Figure 4F and G). It is an interesting observation that ANGPTL3 interacted with the extracellular N-terminus of IL1R1. Since both proteins have signal peptides and undergo similar secretory pathways of protein sorting, which comprises endoplasmic reticulum (ER), Golgi apparatus, and endosomal system such as late endosome and multivesicular body (MVB) (Viotti, 2016). Our hypothesis was that ANGPTL3 may interact with the N-terminal domain of IL1R1 during protein sorting. To investigate this, we examined the co-localization of ANGPTL3 with the full-length IL1R1 or the N-terminal 1–360 aa of IL1R1 at these organelles/vesicles. The results showed that ANGPTL3 and IL1R1 co-localized with markers of ER, Golgi apparatus, late endosome, and MVB (i.e. GFP-Sec61β, YFP-GalT, Rab7-YFP, and CD63-GFP, respectively), but ANGPTL3 and IL1R1 did not co-localize at lysosomes, which are marked by LAMP1-GFP (Supplementary Figure S1A). Similarly, ANGPTL3 partly co-localized with the N-terminal 1–360 aa of IL1R1 at ER, Golgi apparatus, late endosome, and MVB (Supplementary Figure S1B and C). These results suggested that ANGPTL3 co-localized with IL1R1 or its N-terminal region during the protein sorting. Collectively, these results suggested that ANGPTL3 targeted the IL1R1–IL1RAP-associated complex to regulate IL-1β-triggered signaling, and both N- and C-terminal regions of IL1R1 and IL1RAP interacted with ANGPTL3.

### Intracellular FLD domain of ANGPTL3 is involved in the negative regulation of IL-1β-induced signaling

**Figure 5:**
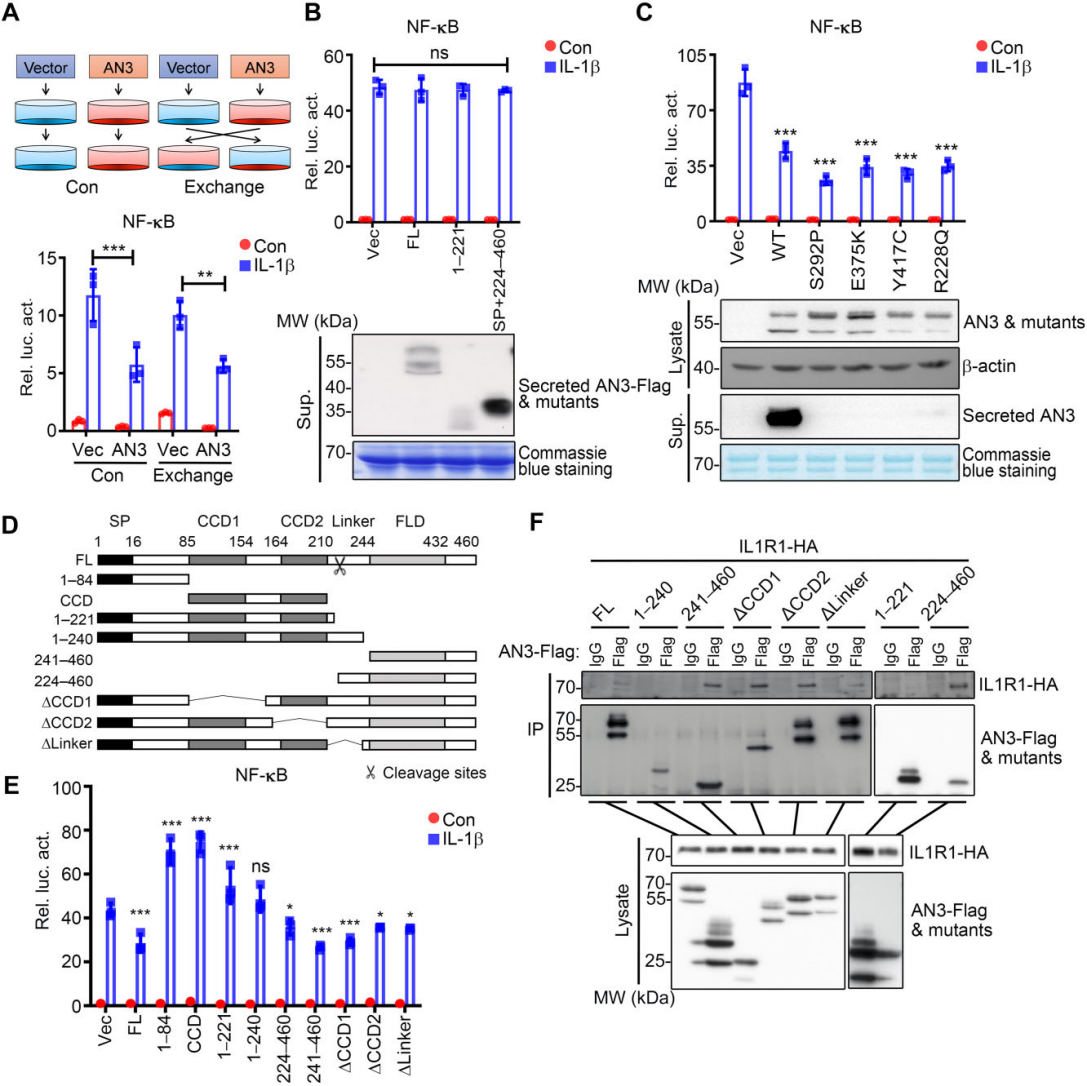
The intracellular FLD domain of ANGPTL3 is involved in the negative regulation of IL-1β-induced signaling. (**A**) The effects of intracellular and secreted ANGPTL3 on IL-1β-induced NF-κB activation were determined by the reporter assay in HEK293T cells. (**B**) The effects of secreted full-length or truncated ANGPTL3 on IL-1β-induced NF-κB activation were determined by the reporter assay and western blotting in HEK293T cells. (**C**) The effects of wild-type or secretion-defective mutants of ANGPTL3 on the regulation of IL-1β-induced NF-κB activation were determined by the reporter assay and western blotting in HEK293T cells. (**D**) A schematic presentation of human ANGPTL3 and its truncation mutants. (**E**) The effects of full-length and various mutants of ANGPTL3 on IL-1β-induced NF-κB activation were determined by the reporter assay in HEK293T cells. (**F**) The interaction of IL1R1 with full-length and truncation mutants of ANGPTL3 was determined by Co-IP in HEK293T cells. AN3, ANGPTL3; Sup., supernatant; FL, full-length; WT, wild-type; SP, signal peptide; CCD, coiled-coil domain; FLD, fibrinogen-like domain. *P < 0.05; **P < 0.01; ***P < 0.001; ns, not significant.

Since ANGPTL3 is a secreted protein and targets the IL1R1-related complex, we further investigated whether secreted ANGPTL3 has similar inhibitory effects on IL-1β-triggered signaling. In HEK293T cells, we transfected ANGPTL3-Flag or control plasmids for 48 h, then the medium was exchanged for 12 h, followed by the IL-1β stimulation (Figure 5A, top panel). Surprisingly, the medium change did not reverse the inhibitory effects of ANGPTL3 on NF-κB activation as determined by the reporter assay (Figure 5A, bottom panel). Furthermore, the HEK293T cells treated with conditioned medium containing secreted full-length ANGPTL3 or its N- or C-terminal fragments showed no inhibitory effects as well (Figure 5B, top panel), even though these proteins were easily detected in the medium (Figure 5B, bottom panel). S292P, E375K, Y417C, and R228Q are four human clinical mutations of ANGPTL3, which abolish the secretion of ANGPTL3 and are related to lower TG levels in human plasma (Romeo et al., 2009). Consistently, all these mutations have similar inhibitory effects as wild-type ANGPTL3 (Figure 5C, top panel), even in the absence of secretion into the medium (Figure 5C, bottom panel). Collectively, these results indicated that it is the intracellular ANGPTL3 but not secreted ANGPTL3 that inhibits the IL-1β-triggered NF-κB activation.

To further map the regions of ANGPTL3 involved in the regulation of IL-1β-induced signaling, we constructed a set of truncation mutants of ANGPTL3 (Figure 5D). Of the three major ANGPTL3 domains, the N-terminal signal peptide facilitates protein sorting and secretion, CCDs play roles in protein–protein interaction, oligomerization, and inhibition of LPL activity, while the C-terminal FLD shares the highest homology with other ANGPTLs and functions as a ligand of integrin αVβ3 to stimulate endothelial cell adhesion and angiogenesis (Yang et al., 2022). Reporter assay results indicated that overexpression of the N-terminal 1–84 aa region or CCDs potentiated IL-1β-induced NF-κB activation; whereas overexpression of the C-terminus of ANGPTL3, including 224–460 aa and 241–460 aa, all significantly inhibited IL-1β-induced NF-κB activation (Figure 5E). These data indicated that the C-terminal FLD region of ANGPTL3 is important for its inhibitory roles, while the N-terminal region may block these effects (Figure 5E). Domain-mapping experiments were performed to investigate the interaction between IL1R1 and the different regions of ANGPTL3. Consistent with reporter assay results (Figure 5E), the C-terminal regions of ANGPTL3 (224–460 aa and 241–460 aa) showed strong interaction with IL1R1, while the N-terminal regions of ANGPTL3 (1–221 aa and 1–240 aa) showed no interaction with IL1R1 (Figure 5F). Collectively, these results suggested that the intracellular FLD domain of ANGPTL3 was critical for its inhibitory effect on NF-κB activation.

### ANGPTL3 inhibits the formation of IL1R1-associated complex

**Figure 6:**
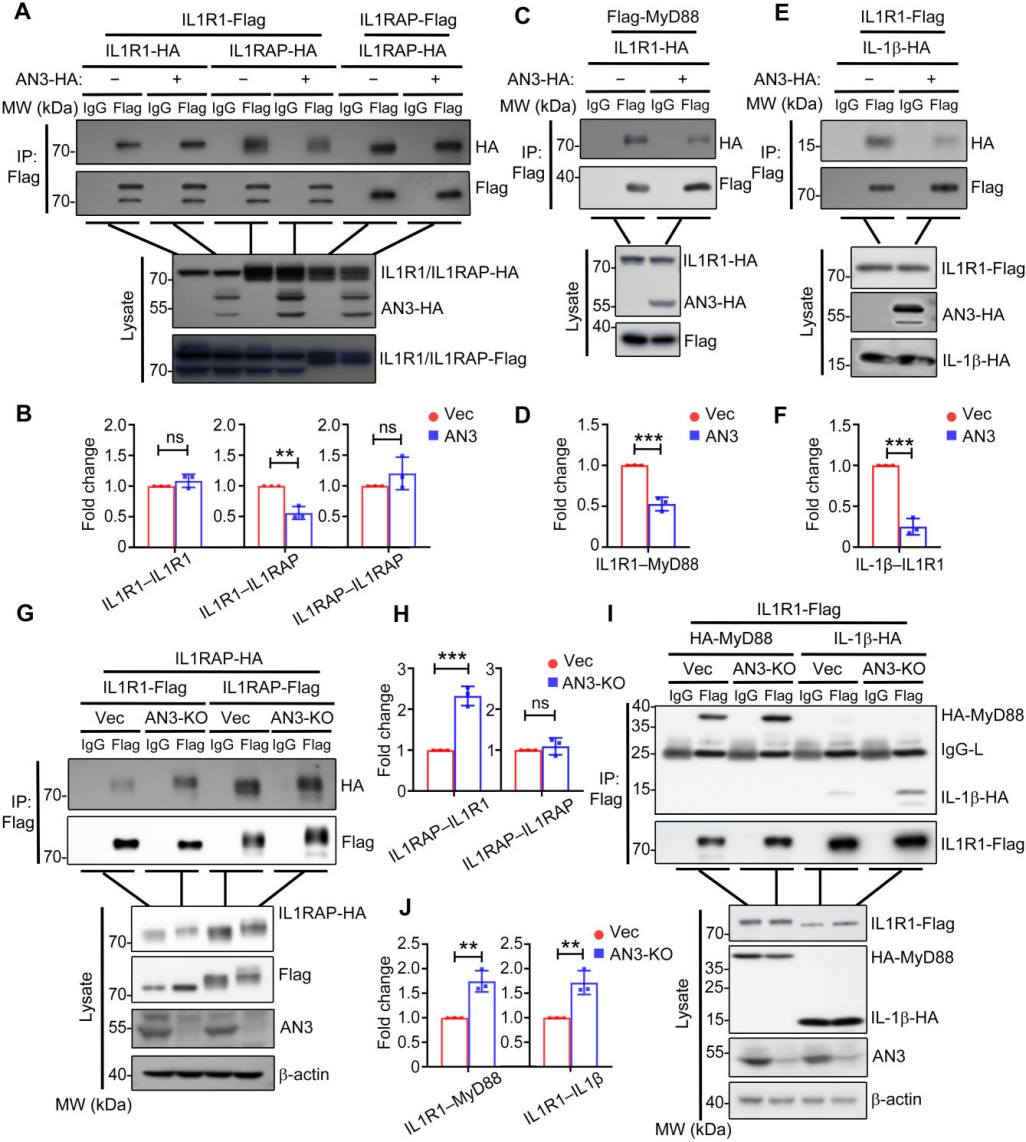
ANGPTL3 inhibits the formation of IL1R1-associated complex. (**A** and **B**) The effects of ANGPTL3 overexpression on IL1R1 or IL1RAP homo- or hetero-interactions were determined by Co-IP (**A**) and quantitative analysis (**B**) in HEK293T cells. (**C**–**F**) The effects of ANGPTL3 overexpression on the IL1R1–MyD88 and IL1R1–IL-1β interactions were determined by Co-IP (**C** and **E**) and quantitative analysis (**D** and **F**) in HEK293T cells. (**G** and **H**) The effects of ANGPTL3 deficiency on the IL1R1–IL1RAP and IL1RAP–IL1RAP interactions were determined by Co-IP (**G**) and quantitative analysis (**H**) in HepG2 cells. (**I** and **J**) The effects of ANGPTL3 deficiency on the IL1R1–MyD88 and IL1R1–IL-1β interactions were determined by Co-IP (**I**) and quantitative analysis (**J**) in HepG2 cells. Quantification results were based on at least three experimental repeats. AN3, ANGPTL3; KO, knockout. **P < 0.01; ***P < 0.001; ns, not significant.

Since ANGPTL3 targets the IL1R1-associated complex, we explored whether ANGPTL3 affects the assembly of this complex by using a Co-IP experiment. ANGPTL3 specifically inhibited the association between IL1R1 and IL1RAP (Figure 6A and B); however, ANGPTL3 did not affect the IL1R1–IL1R1 or IL1RAP–IL1RAP interactions (Figure 6A and B). Since the assembly of IL1R1–IL1RAP hetero-oligomer is critical for its recognition of IL-1β and the recruitment of MyD88 (Lin et al., 2010; Boraschi et al., 2018), we further detected whether ANGPTL3 regulated the IL-1β recognition and MyD88 recruitment by IL1R1. Consistently, we found that the IL1R1–MyD88 and IL1R1–IL-1β interactions were attenuated by the overexpression of ANGPTL3 as well (Figure 6C–F). In contrast, compared to the controls, ANGPTL3 deficiency greatly promoted the interaction between IL1R1 and its co-receptor IL1RAP (Figure 6G and H). Furthermore, ANGPTL3 deficiency also markedly enhanced the recognition of IL-1β or recruitment of MyD88 by IL1R1 (Figure 6I and J). Whereas, the level of IL1RAP self-assembly was comparable in wild-type and ANGPTL3 knockout cells (Figure 6G and H). These results suggested that ANGPTL3 inhibits IL-1β-signaling via inhibiting the assembly of the IL1R1-associated complex.

### Intracellular ANGPTL3 inhibits the IL1R1–IL1RAP interaction to attenuate the IL-1β-induced signaling

**Figure 7:**
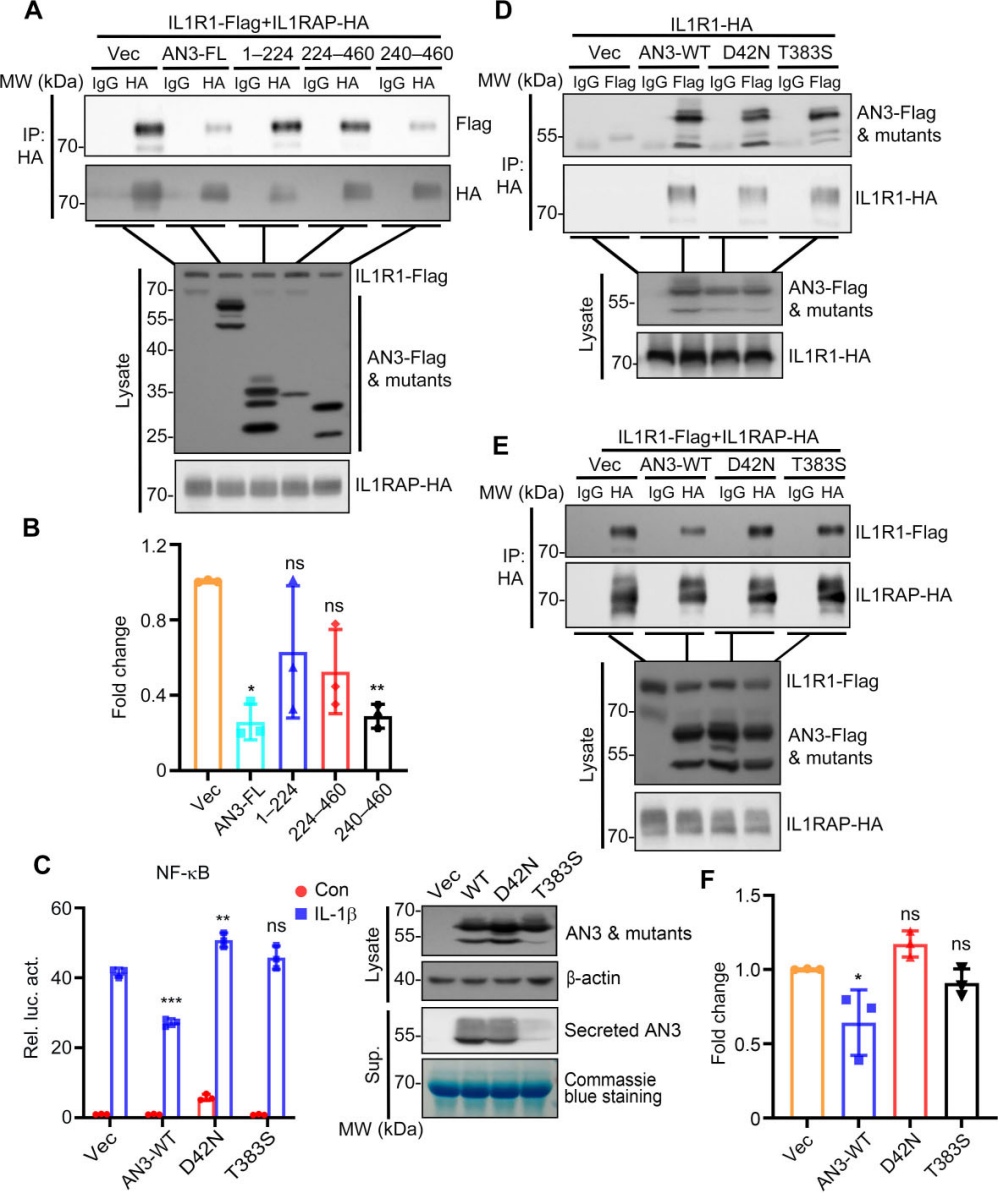
Intracellular ANGPTL3 inhibits the IL1R1–IL1RAP interaction to attenuate IL-1β-induced signaling. (**A** and **B**) Full-length ANGPTL3 and its truncation mutants were overexpressed in HEK293T cells. The effects of the full-length ANGPTL3 and its truncation mutants on the IL1R1–IL1RAP interaction were determined by Co-IP (**A**) and quantitative analysis (**B**). (**C**–**F**) Wild-type ANGPTL3 and its loss-of-function mutants were overexpressed in HEK293T cells. (**C**) The effects of wild-type and mutant ANGPTL3 on the regulation of IL-1β-induced NF-κB activation were determined by the reporter assay (left panel). The expression levels of Flag-tagged wild-type and mutant ANGPTL3 in cell lysate and supernatant were detected by western blotting (right panel). (**D**) The interaction of overexpressed IL1R1 with wild-type and mutant ANGPTL3 was determined by Co-IP. (**E** and **F**) The effects of wild-type and mutant ANGPTL3 on the IL1R1–IL1RAP interaction was determined by Co-IP (**E**) and quantitative analysis (**F**). Quantification results were based on at least three experimental repeats. AN3, ANGPTL3; FL, full-length; Sup., supernatant; WT, wild-type. *P < 0.05; **P < 0.01; ***P < 0.001; ns, not significant.

We further examined the functional consequences of the interaction between ANGPTL3 and IL1R1 protein complexes. FLD is the domain critical for ANGPTL3-facilitated inhibition of IL-1β-induced inflammation and IL1R1 association (Figure 5E and F). Consistently, we confirmed that it is the FLD domain that attenuates the IL1R1–IL1RAP interaction (Figure 7A and B). In contrast, the two clinical loss-of-function ANGPTL3 mutants, D42N and T383S (Stitziel et al., 2017), which could not inhibit IL-1β-triggered NF-κB activation (Figure 7C), were unable to inhibit the assembly of the IL1R1–IL1RAP complex (Figure 7E and F), although they retained the capacity to interact with IL1R1 (Figure 7D). Collectively, these results suggested that inhibition of the IL1R1–IL1RAP interaction is an important mechanism for ANGPTL3-mediated attenuation of IL-1β-triggered signaling.

Taken together, our study clearly demonstrated that ANGPTL3 interacted with IL1R1 and IL1RAP both at the resting stage and under IL-1β treatment (Figure 4C). Furthermore, the intracellular but not secreted form of ANGPTL3 exhibited inhibitory effects on inflammation (Figure 5A–C). These findings suggest that ANGPTL3 physically interacts with IL1R1 and IL1RAP to inhibit the formation of IL1R1-related protein complex, thus maintaining immune tolerance and liver homeostasis at the resting stage.

## Discussion

IL-1β–IL1R1-mediated NF-κB activation and inflammation are crucial mechanisms in the amplification of the inflammatory response, which is tightly controlled to avoid tissue/organ damage. Consistently, antagonists of IL-1 signaling, such as IL1Ra (anakinra), anti-IL-1β mAb (canakinumab), anti-IL-1α mAb (MABp1), and a fusion protein consisting of the ligand-binding regions of IL1R1 and IL1RAP linked to the Fc region of human IgG1 (rilonacept), not only work as inhibitors of infectious or non-infectious inflammatory diseases (e.g. COVID-19 and arthritis), but also serve as promising therapeutic strategy in many chronic inflammatory diseases, such as diabetes mellitus, obesity, cardiovascular diseases, malignancies, and so on (Khazim et al., 2018; Garlanda and Mantovani, 2021).

The liver is a central metabolic organ, which constantly exposed to exogenous proteins or microbiota, and it is also enriched with innate immune cells, making finely regulated IL-1β–IL1R1 signaling critical for maintaining liver homeostasis (Heymann and Tacke, 2016). Studies have shown that deletion of IL1R1 in hepatocytes or macrophage leads to the alleviation of liver inflammation, steatosis, and damage in mice, and the use of IL1Ra benefits patients with severe alcoholic steatohepatitis (Szabo et al., 2022). ANGPTL3, which is mainly expressed in the liver, is a key regulator in TG metabolism. The mAb and other inhibitors of ANGPTL3 have been approved by the FDA or are under clinical trials for the treatment of dyslipidemia and atherosclerosis (Yang et al., 2022). Here, we report that ANGPTL3 inhibits IL-1β-induced NF-κB activation via sustaining the assembly of the IL1R1-associated complex. Notably, the ANGPTL3–IL1R1 and ANGPTL3–IL1RAP interactions could be detected under either the resting stage or IL-1β stimulation (Figure 4C). However, two clinical loss-of-function ANGPTL3 mutants, D42N and T383S, which lost their ability to inhibit IL-1β-triggered NF-κB activation, were unable to inhibit the assembly of the IL1R1–IL1RAP complex (Figure 7C–F). These results imply that such inhibitory roles of ANGPTL3 exist under both physiological and pathological conditions, which may be important in maintaining liver fitness and homeostasis.

It should be noted that although ANGPTL3 is considered mainly expressed in the liver/hepatocytes, it is also detected and functions in other tissues/cells. For example, ANGPTL3 has been detected in the kidney, and has been found to contribute to the enhanced cellular apoptosis and sorafenib response in renal cell carcinoma by inhibiting the focal adhesion kinase (Bao et al., 2018); while *Angptl3^−^^/^^−^* protected against glomerulosclerosis in adriamycin-induced nephropathy by attenuating podocyte loss (Dai et al., 2019). Here, the upregulation of inflammatory genes upon ANGPTL3 deficiency was also observed in THP1 and HEK293T cells (Figures 2F–H, 3D and E), suggesting that similar inhibitory effects mediated by ANGPTL3 may exist in other tissues or cell types such as kidney or myeloid cells. Notably, the liver has a high density of myeloid cells, for example, the local resident macrophage of the liver, termed Kupffer cells, harbors ∼80% of the body's tissue macrophage; moreover, the liver is also patrolled by infiltrating monocyte-derived macrophages (Heymann and Tacke, 2016). Liver homeostasis is orchestrated by the crosstalk between the resident or infiltrating myeloid cells and hepatocytes (Weston et al., 2019). Therefore, future investigation into whether ANGPTL3 plays similar roles in primary myeloid cells would be of great interest.

The ANGPTL family consists of eight secreted members, termed ANGPTL1–ANGPTL8. Among them, ANGPTL4 and ANGPTL8 can form a dynamic complex with ANGPTL3 to cooperatively inhibit the activity of LPL, which is termed the ‘ANGPTL3–4–8 triad’ (Zhang and Zhang, 2022). Knockout of ANGPTL4 or ANGPTL8 in mice or treatment with mAb targeting these proteins induced a favorable lipid profile and reduced cardiovascular risk (Morelli et al., 2020). Moreover, genetic evidence suggests that protein mutants/variants of ANGPTL4 or ANGPTL8 are related to the risk of hypolipidemia (Dewey et al., 2016; Helkkula et al., 2021). However, it is important to note that besides their roles in lipid metabolism, ANGPTLs also function as regulators of inflammation, which poses a challenge for the development of ANGPTLs-based drugs and therapies. For example, monkeys treated with ANGPTL4 mAb or mice with *Angptl4* deficiency have been found to exhibit decreased plasma TG levels. At the same time, it has also been observed that these animals develop severe mesenteric lymphadenopathy, which is caused by fatty acid-induced inflammation in mesenteric lymph node macrophages (Lichtenstein et al., 2010; Dewey et al., 2016; Oteng et al., 2017). Besides, hematopoietic cell-specific ANGPTL4 deficiency also increased both foam cell formation and atherosclerosis (Aryal et al., 2016). These studies suggest the crucial role of ANGPTL4 in inflammation and indicate that potential therapies targeting ANGPTL4 to lower lipid levels should be tissue-specific to avoid adverse inflammatory effects, which have important clinical implications. Similarly, ANGPTL8 is also considered a regulator of inflammation. For example, we previously reported that the level of circulating ANGPTL8 is enhanced in patients with acute inflammation, and the intracellular ANGPTL8 facilitates the p62-mediated selective autophagic degradation of IKKγ, leading to the inhibition of NF-κB activation (Zhang et al., 2017). On the other hand, another study suggested that ANGPTL8 is a proinflammatory factor that accelerates liver fibrosis through its receptor LILRB2 and downstream ERK signaling pathways in the context of high fat diet-induced inflammatory activity (Zhang et al., 2022). All these studies help the development of ANGPTL8-related therapies.

Among the three ANGPTL proteins mentioned, ANGPTL3 is currently the most promising candidate as a drug target. Here, we reported the anti-inflammatory role of ANGPTL3. Notably, by using conditioned medium containing secreted ANGPTL3, we found that such effects were mainly mediated by intracellular ANGPTL3. We also noted that the FDA-approved mAb of ANGPTL3 targets circulating ANGPTL3 without affecting the intracellular role of ANGPTL3, while many other ANGPTL3 targeting strategies, such as the antisense oligonucleotides or CRISPR–Cas9-based gene editing targeting both intracellular and extracellular ANGPTL3 (Yang et al., 2022). Thus, our findings revealed a previously unseen intracellular function of ANGPTL3, which highlights the need for further investigations in this area.

As a secreted protein, ANGPTL3 undergoes intracellular and extracellular cleavage at positions Arg^221^↓Ala^222^ and Arg^224^↓Thr^225^, yielding an N-terminal fragment containing two CCDs and C-terminal fragments mainly composed of FLD (Essalmani et al., 2013). The released N- or C-terminus of ANGPTL3 appear to have distinct activities. For example, the N-terminus of ANGPTL3 is important for regulating lipid metabolism, while the C-terminus acts as a ligand of integrin αVβ3, promoting endothelial cell adhesion and angiogenesis (Yang et al., 2022). Here, we have found that the intracellular C-terminal FLD is critical for inhibiting the association between IL1R1 and IL1RAP, thereby reducing IL-1β-triggered inflammation. Interestingly, the N-terminal CCDs exhibits antagonistic effects for such inhibition, indicating a delicate regulation mechanism behind ANGPTL3 activities.

In summary, our study uncovers a new regulatory mechanism involving intracellular ANGPTL3, which inhibits the assembly of IL1R1-associated receptor complex, leading to the inhibition of IL-1β-induced NF-κB activation. This regulation likely contributes to the fine-regulation of inflammation and immune response to sustain liver homeostasis. Given that ANGPTL3 is already a therapeutic target of dyslipidemia, it is reasonable to consider as future directions for questions including whether such regulation also applies to primary myeloid cells in the liver and whether it contributes to liver homeostasis under physiological or pathological settings in both mouse models and patients with hypertriglyceridemia and associated cardiovascular diseases. Future studies utilizing *Angptl3* knockout or transgenic mice and clinical samples will provide valuable insights into these interesting and significant questions. Therefore, our in-depth investigations focusing on the different roles of intracellular and extracellular forms, as well as N-terminal and C-terminal regions, of ANGPTL3 will contribute to the development and evaluation of ANGPTL3-related drugs and therapies.

## Materials and methods

### Reagents, antibodies, and cell lines

Antibodies against IL1R1 and IL1RAP were kindly gifted by Dr Hong-Bing Shu at Wuhan University. Antibodies against ANGPTL3 (R&D Systems, AF3829), MyD88 (Cell Signaling Technology, 4283), Flag tag (Sigma, F1804), HA tag (Sigma, H3663), and Myc tag (Cell Signaling Technology, 2276) were purchased. HEK293T, Hep3B, and THP1 cells were purchased from the China Center for Type Culture Collection (CCTCC), and HepG2 cells were purchased from Procell Biotech.

### Constructs

Plasmids encoding ANGPTL3 were kindly gifted by Dr Yan Wang at Wuhan University. Reporter plasmids for NF-κB and thymidine kinase (TK) were kindly gifted by Dr Hong-Bing Shu at Wuhan University. Mammalian expression plasmids for IL1R1, IL1RAP, and their truncation mutants, as well as MyD88, IL-1β, IRAK1, and IRAK4, were constructed following standard procedures. For the shRNA or sgRNA plasmids of ANGPTL3, double-strand oligonucleotides targeting different regions of the coding sequence of ANGPTL3 were cloned into pSuper plasmids (Oligoengine) or pLenti-CRISPRV2, respectively. The target sequences of human ANGPTL3 are as follows: shANGPTL3 #1 (606–624 bp of the coding sequence) GCTCAGAAGGACTAGTATT; shANGPTL3 #2 (835–853 bp of the coding sequence) GGTAGTCCATGGACATTAA; sgANGPTL3 #1 (892–911 bp of the coding sequence) ACGTGGGAGAACTACAAATA; sgANGPTL3 #2 (174–193 bp of the coding sequence) AGACTTTGTCCATAAGACGA.

### Retrovirus-mediated stable RNAi or knockout cell lines

The stable RNAi or knockout cell lines were constructed as we previously described (Wang et al., 2017; Zhou et al., 2020a). Briefly, the shRNA or sgRNA plasmids of ANGPTL3 were co-transfected with helper plasmids into the packing cell line HEK293T. After 24–30 h of transfection, the medium was collected after passing through a 0.45 μm filter and used to infect target cells, including Hep3B, HepG2, and THP1 cells, in the presence of polybrene (Millipore, TR-1003-G, 6 μg/μl). The infection was repeated twice before the target cells were selected via puromycin (Ameresco, J593, 1 μg/ml) for 48 h.

### Evaluation of the knockdown efficacy of shRNA or sgRNA

Two sgRNAs and two shRNAs targeting different regions of the coding sequence of ANGPTL3 were designed, and the knockdown efficiency of these sgRNAs and shRNAs were evaluated by *in vitro* (Eki et al., 2020) and *in vivo* (Ran et al., 2013) systems as previously reported (Luo et al., 2016; Xu et al., 2022). Briefly, for the *in vitro* system, an ANGPTL3-Flag expressing plasmid was co-transfected with ANGPTL3 sgRNA or shRNA into HEK293T cells, with HA-lacZ acting as a transfection control. Forty-eight hours later, the cells were lysed, and western blotting was performed with an anti-Flag antibody to test the knockdown efficiency of the transfected Flag-tagged ANGPTL3. For the *in vivo* system, we generated ANGPTL3-deficient Hep3B cells with a standard CRISPR–Cas9 or RNAi approach, and the expression of endogenous ANGPTL3 in the stable cell lines was detected with an anti-ANGPTL3 antibody.

### Luciferase reporter assay

The expression plasmids combined with the NF-κB reporter plasmid and the TK reporter plasmid (served as an internal control) were co-transfected into cells (∼5 × 10^4^). After 24–48 h of transfection, the cells were treated with IL-1β (20 ng/ml) for 8 h before they were lysed. The reporter assay was performed with a dual-specific luciferase assay kit (Promega, E1960) as previously described (Liu et al., 2020; Wang et al., 2022). For the medium exchange experiment, the ANGPTL3-Flag or control plasmids were transfected into HEK293T cells (∼5 × 10^4^). Forty-eight hours later, the medium was exchanged for 12 h, after which the cells were stimulated with or without IL-1β for another 8 h before they were harvested for the reporter assay. The relative luciferase activity was calculated as the ratio of NF-κB reporter activity to that of the internal control.

### Preparation of conditioned medium

To prepare the conditioned medium for the reporter assay, HEK293T cells (∼5 × 10^6^) were transfected with the indicated control or expression plasmids for 48 h, and then the medium of the transfected cells was collected and filtered through a 0.22 μm filter.

### Isolation of primary hepatocytes

Primary hepatocytes were isolated by collagenase digestion as we previously described (Sun et al., 2020; Zhang et al., 2020). Briefly, the mouse liver was perfused *in situ* with pre-perfusion buffer and enzyme buffer containing 0.1 mg/ml collagenase (Sigma, C0130) and 2.5 M CaCl_2_, and the cells were passed through a 100-μm mesh and centrifuged at 40× *g* for 5 min. Then, the cells were washed with high-glucose DMEM (Cytiva) three times before being seeded into a 6-well plate (1 × 10^6^ per well).

### qPCR

Total mRNA was extracted from cells with RNAiso (TaKaRa, 9109) and subjected to qPCR analysis. The mRNA levels of target genes were normalized to 18S rRNA. Primers used in this study are listed in Table 1.

**Table 1:** Primers used in this study.

	**Sequence (5′–3′)**
Human *ANGPTL3*	Forward: TCAACTGTCCAGAGGGTTATTCA
	Reverse: CCATTTAGGTTGTTTTCTCCACACT
Human *TNFA*	Forward: ATCCTGGGGGACCCAATGTA
	Reverse: AAAAGAAGGCACAGAGGCCA
Human *CXCL2*	Forward: CAAGAACATCCAAAGTGTGA
	Reverse: CCATTCTTGAGTGTGGCTAT
Human *IL8*	Forward: GAGAGTGATTGAGAGTGGACCAC
	Reverse: CACAACCCTCTGCACCCAGTTT
Murine *ANGPTL3*	Forward: AGCAAGACAACAGCATAAGAGAACTC
	Reverse: TGAGCTGCTTTTCTATTTCTTTTATCTG
Murine *TNFA*	Forward: GGTGATCGGTCCCCAAAGGGATGA
	Reverse: TGGTTTGCTACGACGTGGGCT
Murine *CXCL2*	Forward: ACGGAAGAACCAAAGAGAA
	Reverse: AAATAAGTGAACTCTCAGACAGC
Murine *IL8*	Forward: TTGGAGCCAAGGCAAGAACA
	Reverse: AATGGAGAGGCATCCGGTTC

### Confocal microscopy

HEK293T cells were transfected with the indicated plasmids for 24 h before they were fixed with 4% (*w*/*v*) paraformaldehyde for 15 min at room temperature. The nuclei were stained with DAPI (Sigma, 28718-90-3, 1 μg/ml), and the cells were imaged with a Zeiss LSM780 or Nikon AX confocal microscopy as previously described (Chen et al., 2017; Yang et al., 2021).

### Co-IP

Transfected HEK293T cells (∼5 × 10^6^) or HepG2 cells (∼1 × 10^7^) were lyzed in 1 ml of NP40 lysis buffer (20 mM Tris–HCl (pH 7.4), 150 mM NaCl, 1 mM EDTA (pH 8.0), and 1% NP40) with protease and phosphatase inhibitor cocktails (MCE, HY-10010). The lysate was centrifuged at 12000 rpm for 10 min at 4°C. For each IP, 0.8 ml of the supernatant was incubated with 0.5 μg of the indicated antibody and 25 μl of protein G magnetic beads (Bio-Rad, 161-4023) at 4 °C for 4 h or overnight. The beads were then washed three times with 1 ml of NP40 lysis buffer with 500 mM NaCl. The precipitates were resuspended in 50 μl of SDS loading buffer before western blotting was performed (Chen et al., 2022).

### Statistics

The data were analyzed with GraphPad Prism (version 8). Statistical analysis was performed using two-tailed Student's *t*-test for two experimental groups, and one-way ANOVA for multiple experimental groups without adjustment. Data are reported as the mean values, with error bars showing the standard deviation (SD). At least three independent experiments were performed. A *P-*value <0.05 was considered statistically significant. The full immunoblots are provided in the supplementary information (Supplementary Figures S2–S6).

## Supplementary Material

mjad053_Supplemental_File
