# Supplementary material for: ANGPTL3 negatively regulates IL-1β-induced NF-κB activation by inhibiting the IL1R1-associated signaling complex assembly
Source: J Mol Cell Biol. 2023 Aug 26;15(8):mjad053. doi: 10.1093/jmcb/mjad053 (PMC11149415; doi:10.1093/jmcb/mjad053)
Supplement: mjad053_Supplemental_File [file mjad053_supplemental_file.pdf]

Supplementary material

ANGPTL3 negatively regulates IL-1β-induced NF-κB activation by inhibiting the IL1R1-associated signaling complex assembly

Yu Zhang[1], Zi-tong Zhang[1], Shi-yuan Wan[1], Jing Yang[1], Yu-juan Wei[1], Hui-jing Chen[1], Wan-zhu Zhou[1], Qiu-yi Song[1], Shu-xuan Niu[1], Ling Zheng[2] and Kun Huang[1*]

[1]Tongji School of Pharmacy, Tongji Medical College, Huazhong University of Science & Technology, Wuhan 430030, China

[2]Hubei Key Laboratory of Cell Homeostasis, College of Life Sciences, Wuhan University, Wuhan 430072, China

[*]**Corresponding author**

Kun Huang, Ph.D.

Tongji School of Pharmacy

Huazhong University of Science & Technology

Wuhan 430030, Hubei, P.R. China

E-mail: kunhuang@hust.edu.cn

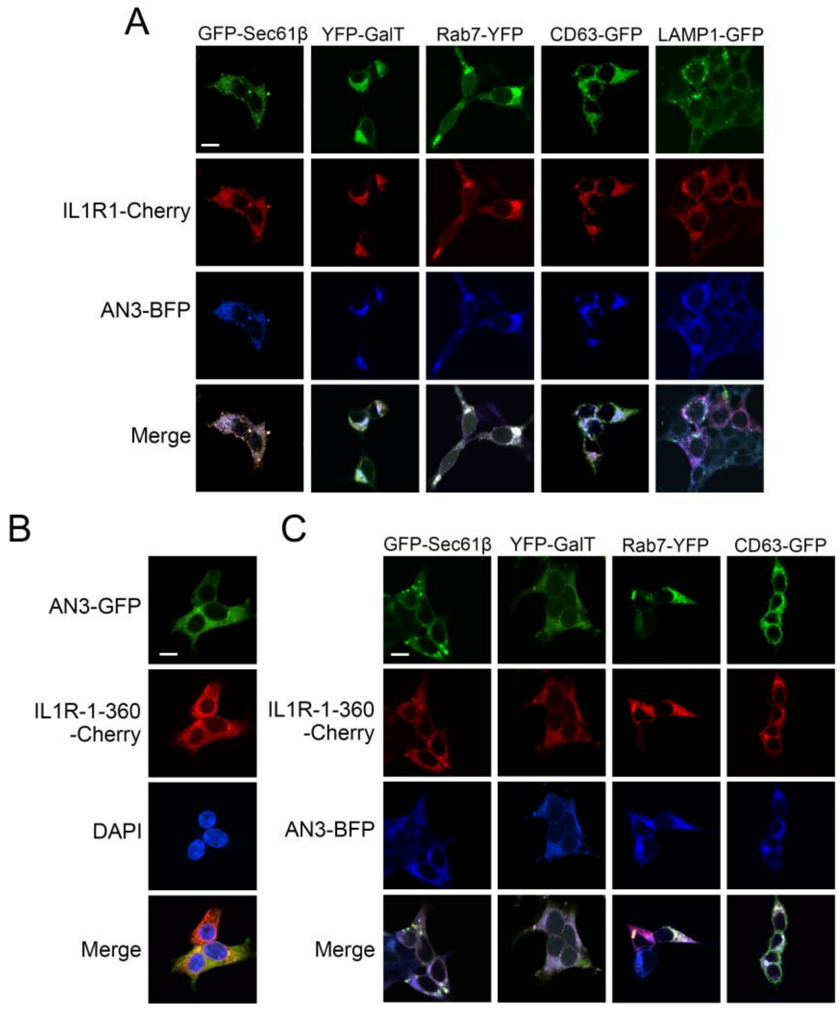

Supplementary Figure S1 Co-localization of ANGPTL3 and full-length or N-terminal of IL1R1 at various cell organelles/vesicles. **(A)** Co-localization between ANGPTL3-BFP, IL1R1-Cherry and markers of ER, Golgi apparatus, late endosome, MVB or lysosome (marked by GFP-Sec61β, YFP-GalT, Rab7-YFP, CD63-GFP or LAMP1-GFP, respectively) in HEK293T cells. The scale bar represents 10 μm. **(B)** Co-localization of ANGPTL3-Cherry and IL1R1-1-360-GFP in HEK293T cells. The scale bar represents 10 μm. **(C)** Co-localization between ANGPTL3-BFP, IL1R1-1-360-Cherry and markers of ER, Golgi apparatus, late endosome or MVB as in (A) in HEK293T cells. The scale bar represents 10 μm.

.

Figure 1

A

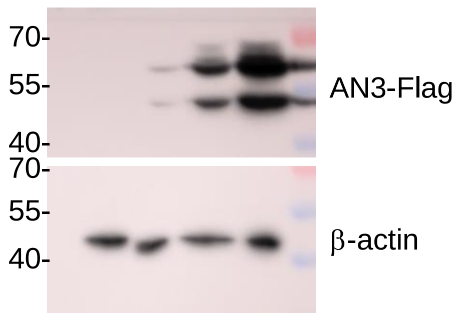

B

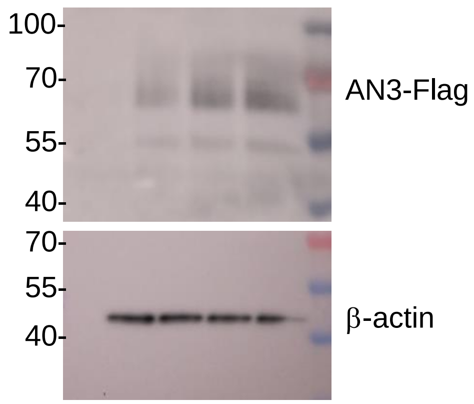

Figure 2

A

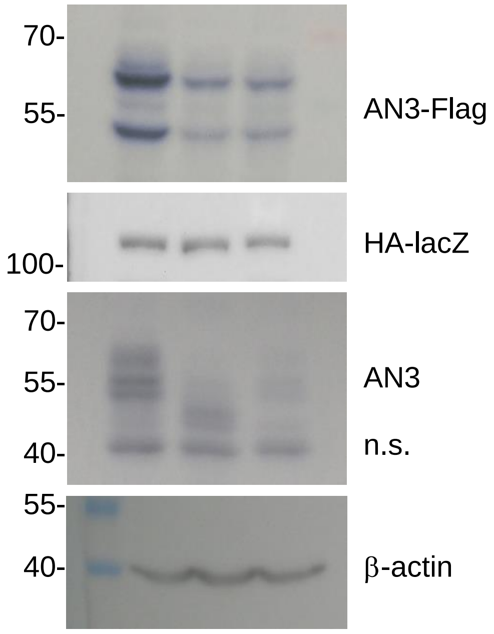

Figure 3

A

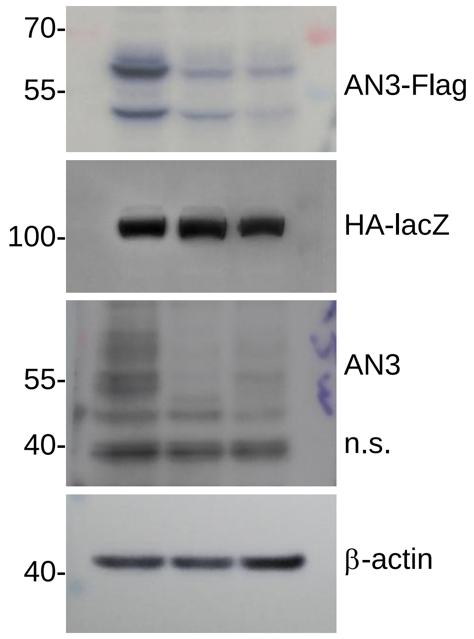

Supplementary Figure S2 The full immunoblots for figure 1 - 3.

Figure 4

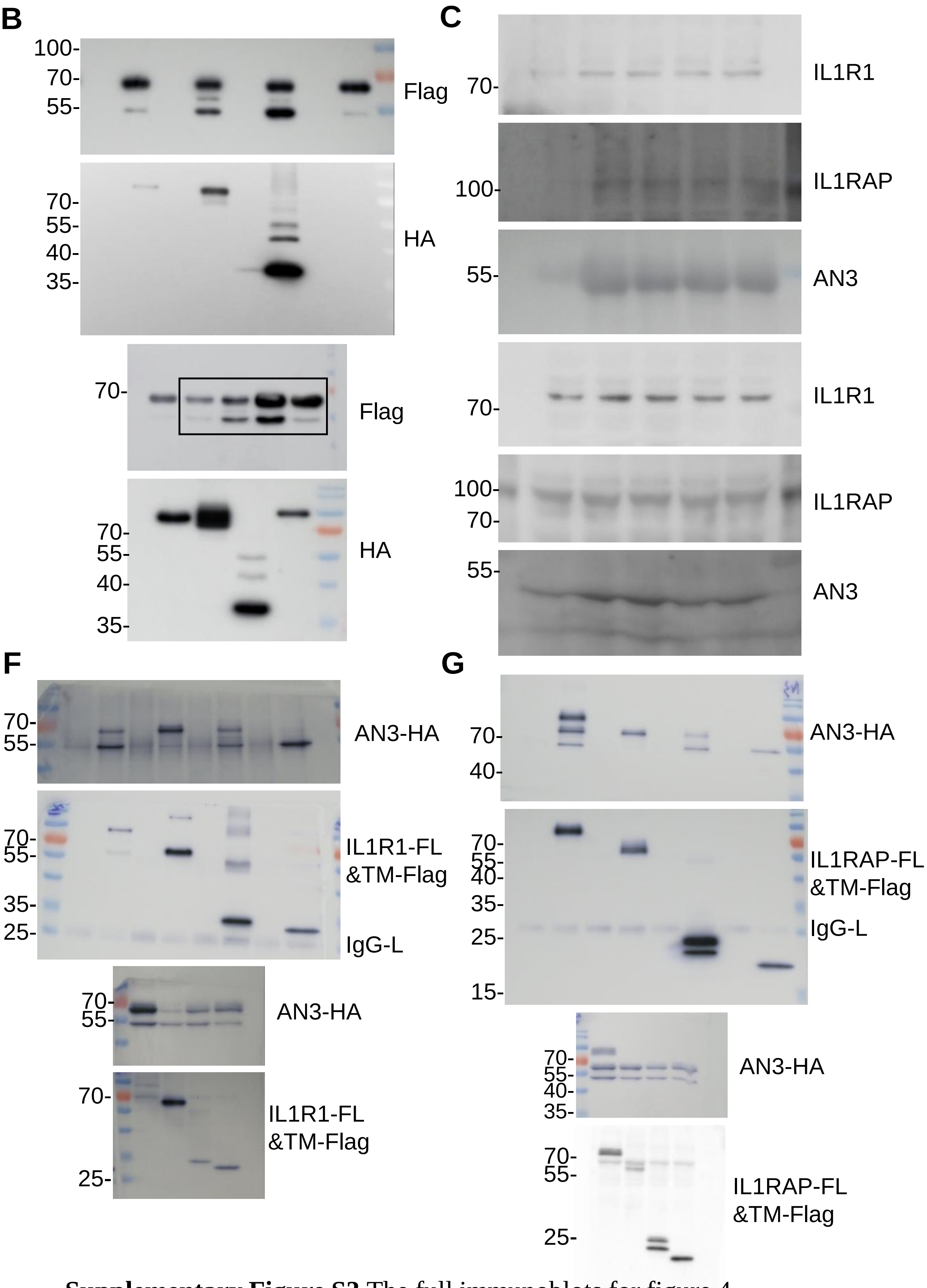

Supplementary Figure S3 The full immunoblots for figure 4.

Figure 5

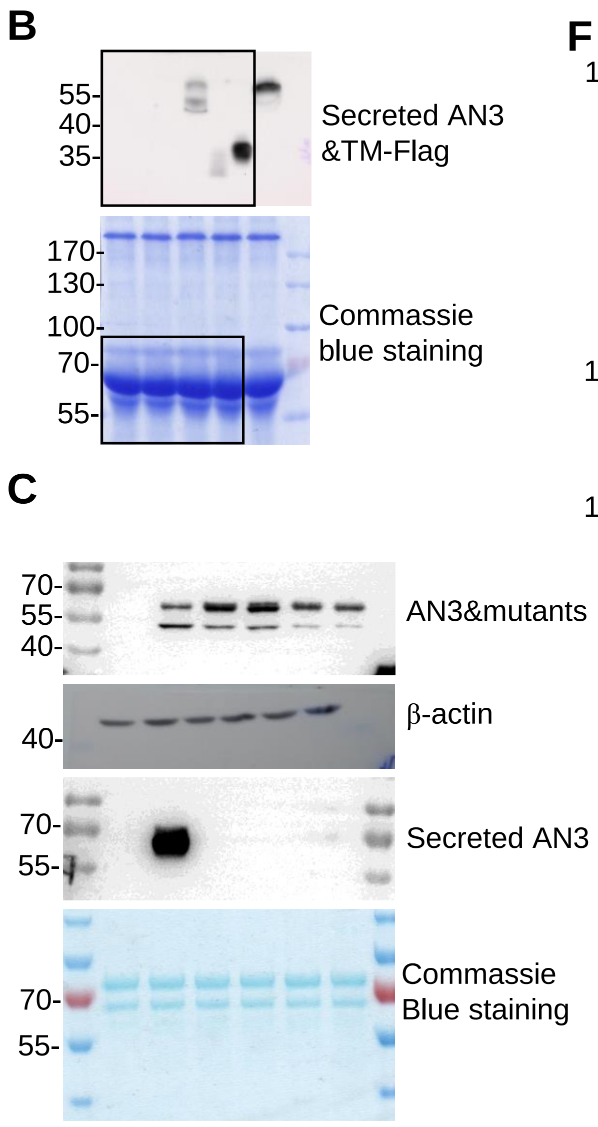

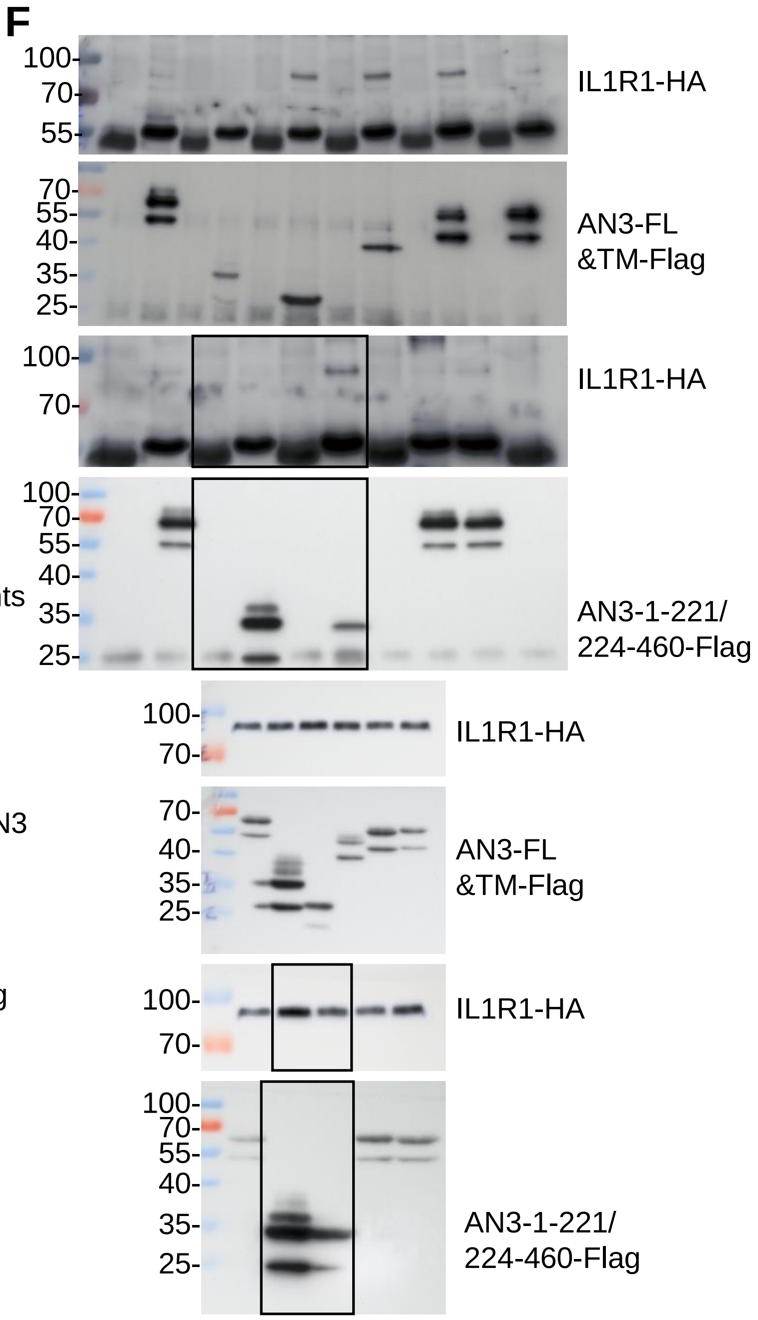

Figure 6

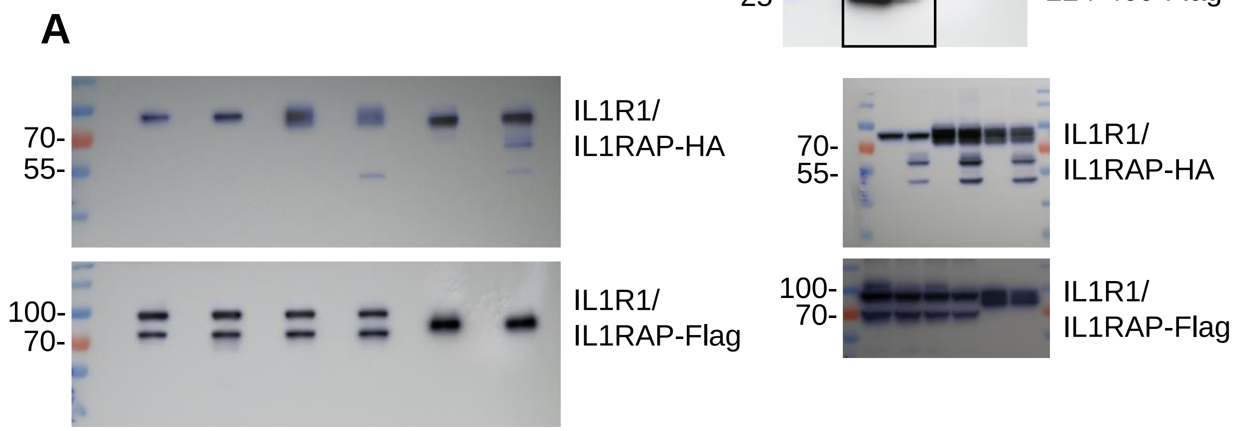

Supplementary Figure S4 The full immunoblots for figure 5 and 6A.

Figure 6

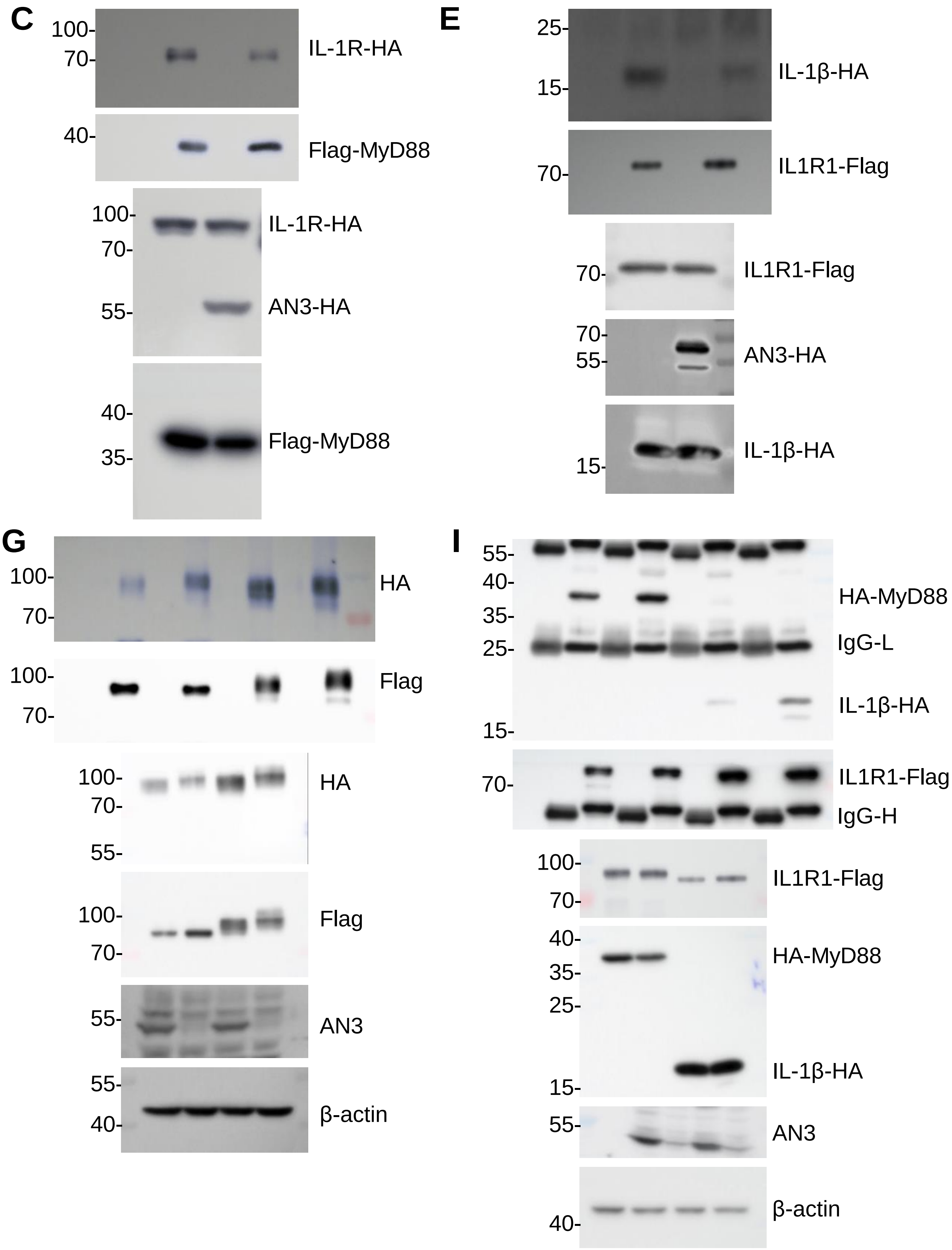

Supplementary Figure S5 The full immunoblots for figure 6C, E, G and I.

Figure 7

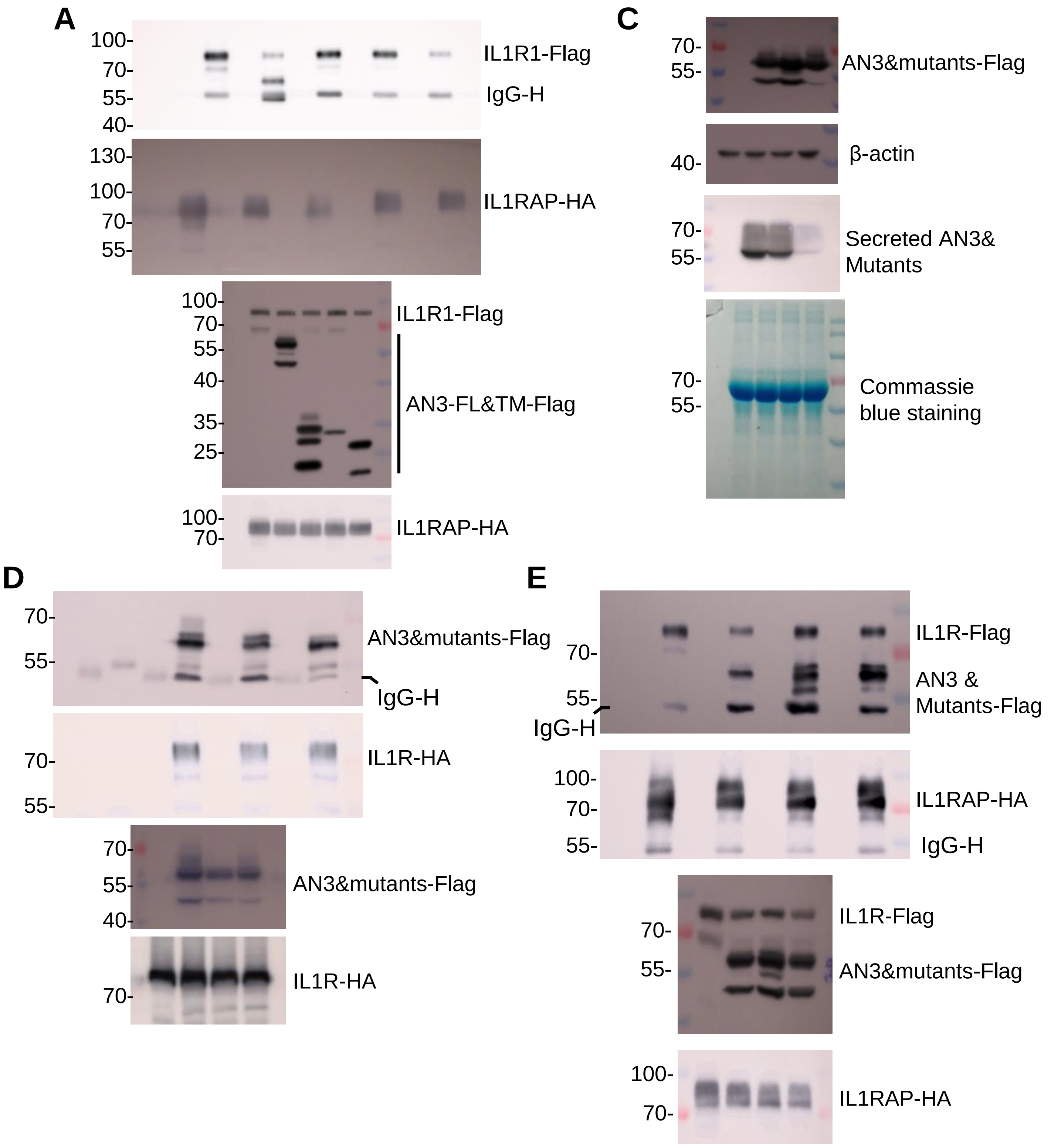

Supplementary Figure S6 The full immunoblots for figure 7.
